# Performance and emission analysis of a CI engine fueled with parsley biodiesel–diesel blend

**DOI:** 10.1007/s40243-022-00213-4

**Published:** 2022-07-22

**Authors:** Sarah Oluwabunmi Bitire, Tien-Chien Jen

**Affiliations:** Department of Mechanical Engineering Science, University of Johannesburg, Auckland Park, Johannesburg, 2006 South Africa

**Keywords:** Parsley biodiesel, Diesel engine, Emissions, Performance, Greenhouse gases

## Abstract

Pollution-induced environmental deterioration is one of the serious aspects that must be solved. As a result, biodiesel was made from a novel material (Parsley seed oil) through an alkali-induced transesterification reaction. The efficiency, as well as exhaust emission tests, were performed by running the prepared parsley biodiesel blends (mixture of biodiesel and diesel fuel in different proportions) in an engine. The ideal blend for enhancing engine performance was discovered to be B20, which displayed steady performance attributes without requiring any modifications to the diesel engine. The B20 parsley biodiesel blend had fewer emissions than diesel, notably hydrocarbons, and carbon monoxide except for nitrogen oxides and carbon dioxide. B20 Parsley blends were also shown to emit less pollution than other blends (B5 and B10). A high reduction in CO, CO_2_ and HC emissions for B20 was recorded at 33.9%, 29.73%, and 11.38% relative to diesel except for NO_*x*_. Brake-specific energy consumption decreases and thermal efficiency of the engine increases for all biodiesel blends. In addition, from the performance results, BTE and BSFC of B20 are relatively close to those of pure diesel fuel (B0). The use of parsley biodiesel as a diesel engine fuel was shown to be a promising strategy to promote the use of green fuels (biofuels from renewable materials) while simultaneously mitigating the release of toxic greenhouse gases from the combustion of fossil fuel.

## Introduction

The existing reliance on fossil fuels is high, despite the fact they produce toxic gaseous emissions which are very harmful when released into the atmosphere [1]. These harmful pollutants have endangered countless lives due to their detrimental effect on the ozone layer which shields the earth from the direct impact of sunlight ultra-violet (UV) radiation [2]. Air pollution resulting from human activities has culminated in poor air quality, particularly in urban areas. According to a 2014 report by the World Health Organization (WHO), air pollution claimed the lives of about seven million urban residents worldwide in 2012 [3]. Moreover, nine out of ten individuals on the planet are incapable of breathing clean air. In addition, the location, where air pollution is extreme and exceeds the limitations are observed to have very detrimental consequences, as such regions have a higher possibility of illness as well as a criticality in patients with COVID-19 than less polluted regions [4]. The accumulation of huge amounts of gaseous pollutants in the atmosphere is putting the human ecology at risk of pollution. As a result, the climate is transforming, air temperatures are increasing owing to the retained heat from amassed gases. The glaciers are melting and prompting a rapid rise in sea levels faster, and gaseous pollution is contributing to severe respiratory ailments and health difficulties [5]. As a result of this urgent necessity, a global transition from the utilization of conventional fuels to the use of renewable energy sources is needed. Moreover, the utilization of renewable resources to generate energy offers tremendous possibilities for eradicating pollution and building a healthy environment.

Many energy-generation processes that promote environmental sustainability are rapidly emerging as part of green technology. One of the most effective approaches to preserving nature and the environment is to produce and use biofuels, especially in the automotive sector. A lot of research studies on the development and implementation of renewable energy have proven that, because of their renewability and environmental friendliness, renewables can effectively substitute fossil fuels. In addition, several new studies providing proactive solutions have been witnessing a high level of a worldwide shift to renewable energy over the years as the world strives to achieve massive generation and trade-off rates of biofuels [6]. Recent studies are devising new feedstock as well as methodologies to make huge success in reducing noxious engine emissions from the burning of fossil fuels as a result of the expanding usage of biofuels in vehicle engines [7]. For instance, biodiesel derived from low-cost feedstock has been revealed to possess clean-combustion features that have the potential to lessen worldwide reliance on diesel when utilized in unaltered vehicles.

Moreover, various studies have reported the employment of biodiesel blends to boost a diesel engine’s efficiency as well as emission qualities. Ogunkunle and Ahmed [8] employed a six-cylinder diesel engine to run on Parinari polyandra biodiesel blends. The authors discovered that the blends utilized improved the engine performance and also reduced emissions of carbon dioxide (CO_2_), hydrocarbon (HC), and carbon monoxide (CO) by about 65.7%, 53.8%, and 81.7%, respectively. Nitrogen oxides (NO_*x*_) emissions, on the other hand, rose by 35.1% showing a negative effect. Elkelawy et al. [9] prepared biodiesel blends of D70B30, D50B50, and D30B70 with a mixture of sunflower and soyabean oil. These prepared test samples were then compared to neat diesel. They noticed that the biodiesel blends had a substantial influence (improved) on the brake specific fuel consumption, and brake thermal efficiency. The emissions of unburnt hydrocarbon and carbon monoxide were reduced by 41.18% and 33.8%, while the emission of oxides of nitrogen was higher than that of diesel fuel. Liaquat et al. [10] examined the performance and emission characteristics of a diesel engine by employing coconut biodiesel and its blends. The study demonstrated that biodiesel blends emitted less CO, CO_2_, and HC as well as a higher NO_*x*_ emission at all loads compared to diesel fuel. In addition, an increase in brake specific fuel consumption by 2.11% was observed for the blend in comparison with diesel fuel. Datta, Palit and Mandal [11], also tested different proportions of biodiesel blends generated from Jatropha biodiesel. The authors reported that the brake thermal efficiency (BTE) of biodiesel blends decreased when the %age of biodiesel in the blend increased, while Brake specific fuel consumption (BSFC) increased as the proportion of biodiesel in the blend increased. In addition, the greenhouse gas emissions were reduced except for NO_*x*_ emissions. Table 1 displays a list of a few related works on the subject. Research investigations continue to indicate that biodiesel fuels made from a variety of seed oils (rubber seed oil, date seed oil, hemp seed oil, cotton seed oil, African pear seed oil, watermelon seed oil, etc.) have fuel qualities comparable to diesel fuel while emitting less hazardous pollutants [12–17]. The utilization of biodiesel produced from seed oils has become increasingly popular as a diesel fuel substitute due to its many characteristics that might enhance the proper functioning of a diesel engine. Nevertheless, there are a number of issues that arises with their use as diesel engine fuel. Some biodiesel has a viscosity that is higher than diesel making it viscous [18]. High viscosity can result in difficulties, such as poor atomization of fuel [18, 19] Also, the density is a fuel characteristic that may have an impact on the use of biodiesel in combustion ignition engines, given that fuel injection systems measure fuels by volume. The engine power output is impacted by differences in density brought on by various fuel masses injected [10]. Therefore, density plays a crucial role in influencing the diesel engine's efficiency. Biodiesel has a mass-based net calorific value that is around 12% lower than diesel, which means it has less energy than diesel and can operate engines at lower speeds and with greater efficiency [20]. Most of these issues related to the direct use of biodiesel in internal combustion engines can be resolved by blending diesel fuel with biodiesel in a variety of ratios. Hence, biodiesel blends with diesel fuel and Parsley biodiesel for sustainable and clean biodiesel generation and the engine uses is currently in its early stages, with a limited research focus. Parsley is a promising energy crop that has not yet reached its full potential. Moreover, the application of parsley biodiesel has not been thoroughly studied to assess its effect on the performance and emission of a diesel engine. To the best of the authors’ knowledge, experimental studies on parsley biodiesel's efficiency as well as emission qualities, which must be based on facts relating to its usage as a green fuel in an engine have not been documented. Parsley is one of Africa's prospective biodiesel feedstock with low fatty acid content. Parsley seeds have the potential for biodiesel synthesis, which is one of the most important variables to be considered when selecting a material for the generation of good-quality biodiesel [21]. Based on the findings of Ramos et al. [22], the fatty acid content is amongst the most important aspects influencing biodiesel quality. Parsley seeds have a wide spectrum of saturated as well as unsaturated fatty acids that may be employed to generate biodiesel. Since parsley biodiesel has comparable fuel characteristics to diesel [23]. The seed of parsley according to [24] contains a variety of fatty acids. There are no research investigations on the utilization of the Parsley biodiesel blend as an engine fuel especially on the emissions characteristics when it burns in diesel engines. The main novelty of this research study is to determine the impact of the parsley biodiesel blend on the performance and emission characteristics of a four-stroke, single-cylinder diesel engine. Therefore, it is vital to find out how much Parsley biodiesel blends emit and how well they operate in a diesel engine. The tests were performed using different parsley biodiesel blends in a diesel engine. It was discovered that when the engine was run on blended fuel, the engine performance improved without requiring any engine modifications. In addition, the performance parameters of the diesel engine with B20 were almost similar to those found when the engine was operating on diesel. Except for NO_*x*_, all exhaust pollutants, notably hydrocarbon and carbon monoxide, and carbon dioxide were determined to be lower than those emitted by diesel.

**Table 1:** Performance and emission characteristics of diesel engines fueled with biodiesel blends

Utilized engine	Fuel type utilized	Reaction condition (rpm)	BTE	BSFC	HC	NO_*x*_	CO	References
4-Cylinder 4-stroke, DI, water-cooled	Kusum biodiesel (B5, B10,B15, and B20)	1000–4000	Increased	Decreased	Reduced	Increased	Reduced	[25]
Six-cylinder 4-stroke, DI	Parinari polyandra biodiesel (B10, B20, and B30)	1200	Increased	Decreased	Reduced	Increased	Reduced	[8]
Single-cylinder 4-stroke, DI, water-cooled	Sunflower and soya bean oil (D70B30, D50B50, and D30B70) biodiesel	1400	Decreased	Increased	Reduced	Increased	Reduced	[9]
Single-cylinder 4-stroke, DI	Coconut biodiesel blends (B5 and B15)	1500–2400	–	Increased	Reduced	Increased	Reduced	[10]
Single-cylinder 4-stroke, DI water-cooled	Jatropha biodiesel (B20)	1500	Increased	Decreased	Reduced	Increased	Reduced	[11]
Single-cylinder 4-stroke, DI, water-cooled	Rapeseed, corn oil, waste cooking oil biodiesel, (B10, B20, and B50)	1500	–	–	Reduced	Increased	Reduced	[26]
Single-cylinder 4-stroke, DI,	Jatropha, Moringa, and palm biodiesel (B20)	1000–3500	–	Increased	Reduced	Increased	Reduced	[27]
1-cylinder 4-stroke, DI, air-cooled	Parsley biodiesel (B5, B10, and B20)	1500	Increased	Decreased	Reduced	Increased	Reduced	This study

## Materials and methods

All chemicals employed in this study are all of the analytical grades and were obtained from Sigma-Aldrich. They include *n*-hexane (95% purity), potassium hydroxide (≥ 85% purity), and methanol (≥ 99.9% purity).

### Analytical methods

Using the solvent extraction method, oil was recovered from parsley seeds. For the conversion of oil to biodiesel, a validated optimized transesterification technique was adopted [7]. In addition, the engine operational properties were analyzed using parsley biodiesel blends in comparison to neat diesel fuel. Simultaneously, the emissions were analyzed and documented.

### Oil extraction and biodiesel production

The seeds of parsley were thoroughly cleaned, sun-dried, and reduced into smaller sizes by employing an electric blender. The ground seeds of parsley were then placed in an extractor for about 5 h before proceeding to remove excess extractant (*n*-hexane) by drying at room temperature for 24 h. The extractant of choice was chosen because of its low cost and great efficiency when compared to other solvents used for oil extraction [7]. For fuel sources with a high amount of free fatty acid, two-step transesterification procedures are recommended to reduce free fatty acid [28]. However, the extracted parsley oil yielded 0.78% free fatty acid, showing that the oil may be turned into biodiesel using direct alkali catalysis of the transesterification process. The viscosity of biodiesel made from plant oils must be lowered, as well as the cetane number and heating parameters. As a result, transesterification is frequently required. These are desirable characteristics for methyl ester derived from plant oils used in motor fuel. Because of its quick rate of reaction and shorter functional chain, methanol was chosen as the preferred solvent. The conversion of Parsley oil was achieved by adopting a bioreactor (product code A-02) with a capacity of 50 mL to 100 L possessing about 500 °C optimum temperature (manufactured in Mumbai, India by Amar equipment Pvty. Ltd). A given ratio of the parsley oil, the catalyst of choice, and methanol was placed into the bioreactor and then heated at 60 °C for 1 h reaction time. These operations were carried out with 1 wt% potassium hydroxide as a catalyst and 9:1 excess methanol: oil ratio for favorable forward reaction kinetics. Thereafter, the content of the bioreactor (produced biodiesel and glycerin) was emptied into a separatory funnel and allowed to settle in terms of their various densities. The biodiesel sample was noticed at the upper layer, while the glycerin sample was observed at the bottom of the funnel. Afterward, the obtained biodiesel was washed severally with de-ionized water, dried, and stored in the sample bottle. The procedure was repeated thrice and the average biodiesel yield was recorded. In addition, the desired parametric condition of choice for manufacturing biodiesel from KOH was established from related literature studies [29, 30] that evaluated and tested the best parametric condition for the transesterification reaction. The produced biodiesel was measured by utilizing Eq. (1):

1 $${\text{Parsley biodiesel yield}}=\frac{{P}_{\mathrm{o}}}{ {P}_{\mathrm{s}}} \times 100\%$$

where *P*_o_ = amount of the produced methyl esters, *P*_s_ = amount of the parsley oil employed.

The distinctive features of parsley seed oil were evaluated using the association of analytical chemists’ recognized test methods. The density of the seed oil was determined using a density bottle, and the viscosity of the seed oil was determined using a rotating viscometer. The pH was estimated using a digital pH meter, while Coronado et al. [31] technique was adopted to obtain the specific gravity. To analyze the methyl esters present in the sample, a gas chromatography–mass spectroscopy test was employed. The detected parsley biodiesel’s chromatogram peaks were compared to chromatogram peaks of standards in the NIST libraries. In addition, standard protocols from the American society for testing and materials (ASTM) were adopted to establish the produced biodiesel's physicochemical qualities. The acid value, cetane number, kinematic viscosity, cloud point, higher heating value, pour point, moisture content, as well as flashpoint, were determined employing biofuel specifications, such as D664, D613, D445, D2500, D93, D97, D2709, and D2015, respectively. Furthermore, the properties of the produced biodiesel and extracted oil were examined by adopting equipment, such as a flash point tester, pH meter, fuel calorimeter, cloud and pour point meter, etc.

### Engine analysis utilizing parsley biodiesel blends

The engine studies were carried out using a Honda single-cylinder, four-stroke, air-cooled direct-injection diesel engine (Honda model GD411, manufactured by Honda motor company in Minato, Tokyo, Japan).

**Fig. 1:**
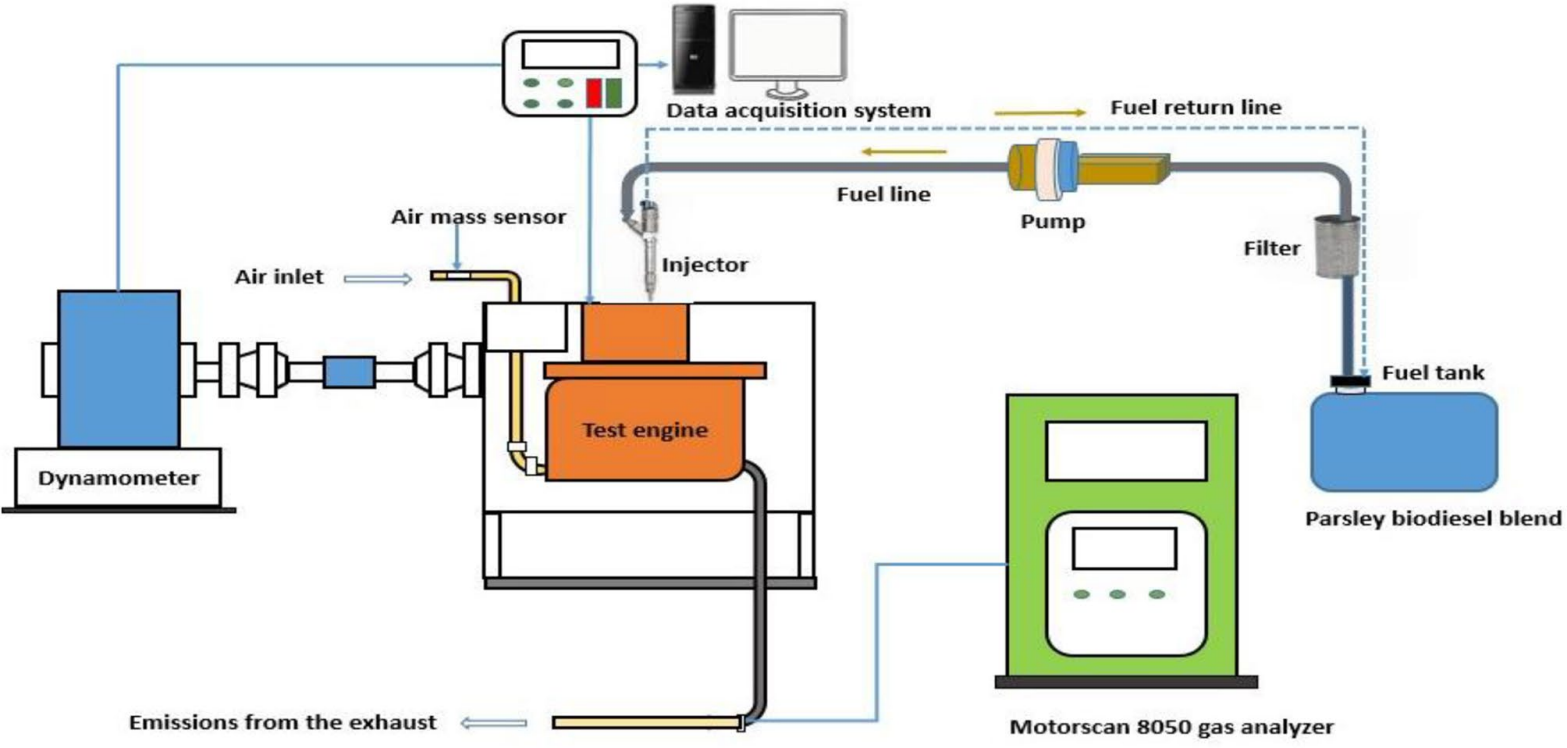
Utilized engine test setup

The engine specifications are listed in Table 2 and a representation of the test engine with all of the essential test equipment is shown in Fig. 1. According to Dharmadhikari, Kumar and Rao [32], biodiesel has a density similar to diesel fuel and so it is possible to blend it directly with diesel. As a result, parsley biodiesel, as well as diesel fuel, were produced in volume ratios of 5:95, 10:90, and 20:80 for B5, B10, and B20, respectively. The engine was operated for 30 min at a steady speed of 1500 rpm using 100% diesel fuel (B0) as the base fuel or reference fuel as described by Rastogi et al. [33]. A dynamometer was utilized to apply the engine load. The fuel consumption was determined using a burette and an eddy current dynamometer under varying loads. The percentage of carbon monoxide (CO), hydrocarbon (HC), and nitrogen oxides (NO_*x*_) emissions from the engine via the exhaust were measured using a total gas analyzer (Motorscan model 8050) made by EOS Motorscan in Parma, Italy. Following the completion of the test with 100% diesel (B0), the engine was set to operate with the biodiesel blends. At a steady speed of 1500 rpm, the findings of the various analysis were recorded. The engine test was conducted by utilizing a modified technique adopted by [9].

**Table 2:** Specification of diesel engine

Parameters	Specifications
Manufacturer	Honda
Number of cylinders	1
Cycle	4-Stoke
Cooling system	Air Cooled
Bore	82.0 mm
Stroke	78.0 mm
Compression ratio	18.2:1
Maximum speed	3750 ± 150 rpm
Injection system	Direct injection
Rated power	9HP (6.6 Kw) at 3600 rpm

## Results and discussion

### Extracted Parsley oil and biodiesel

**Fig. 2:**
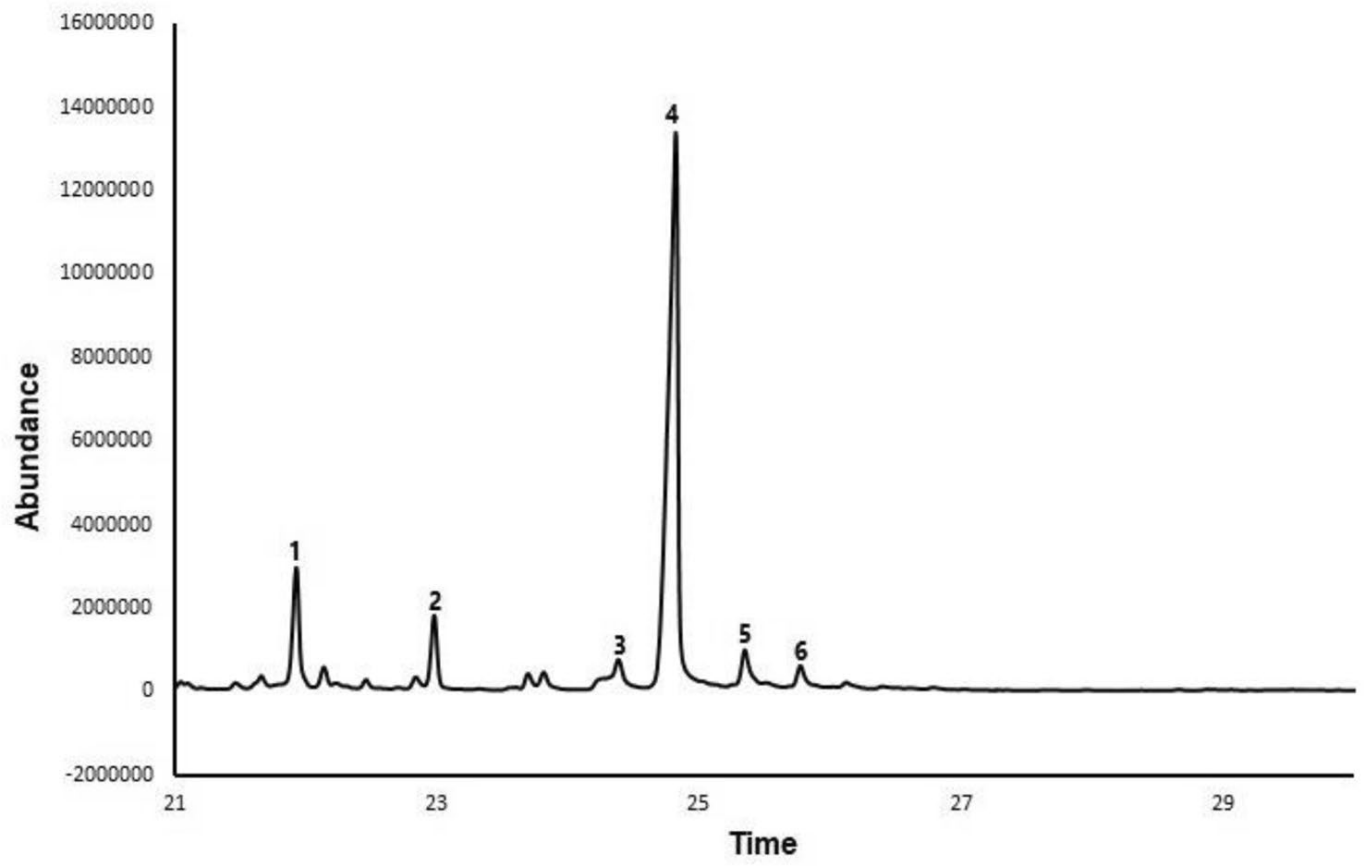
GC–MS chromatogram of biodiesel

The oil recovered from pulverized Parsley seeds had a low free fatty acid content (FFA) of 0.78% according to the findings of Bitire et al. [7]. This signifies that esterification does not have to precede transesterification, since the free fatty acid level is not up to 1%. After extraction, about 25% of the crude oil was recovered. Transesterification of parsley biodiesel yielded an average of 96% biodiesel yield. The available Fatty acid methyl esters (FAME) in the generated biodiesel as measured by Gas chromatography–mass spectrometry (GC–MS) are depicted in Table 3. Table 4 [7] lists the qualities of the extracted oil (PSO) and the acquired chromatogram of parsley biodiesel is shown in Fig. 2. The chromatogram displays six peaks, which correspond to detected esters in the generated biodiesel. Table 4 also contains the average values obtained for biodiesel fuel characteristics (indicated as B100), the attributes of blended fuel samples (B5–B20) as well as that of diesel fuel (B0). The qualities of the blended fuel samples were found to be within the required specification for usage as an engine fuel when assessed in accordance with the American society for testing and materials standards D6751.

**Table 3:** FAME content of parsley biodiesel

Compounds detected	Molecular formula	Composition (%)	#Peaks
Hexadecanoic acid, methyl ester	C_17_H_34_O_2_	12.33	1
11-Octadecenoic acid, methyl ester	C_19_H_36_O_2_	15.07	2
cis-13-Octadecenoic acid, methyl ester	C_19_H_36_O_2_	9.11	3
9,12-octadecadienoic acid, methyl ester	C_19_H_34_O_2_	27.20	4
10-Octadecenoic acid, methyl ester	C_19_H_36_O_2_	1.23	5
Octadecenoic acid, methyl ester	C_19_H_36_O_2_	2.14	6

**Table 4:** Properties of Parsley seed oil, biodiesel blends, and diesel fuel

Fuel properties	PSO	B100	B0	B5	B10	B20
Kinematic viscosity (mm^2^/s) (@ 40 °C)	14.9	5.4	3.02	3.09	3.12	3.20
Saponification value	194.81	–	–	–	–	–
High heating value (kJ/kg)	–	39.54	43.90	43.71	42.76	42.47
Refractive index	1.467		–	–	–	–
Specific gravity (@ 40 °C)	0.991	0.899	0.881	0.890	0.897	0.903
Acid value (mg KOH/g)	1.55	0.43	–	–	–	–
Free fatty acid (%)	0.78		–	–	–	–
Cloud point (°C)	2.1	2.3	− 12.2	− 6.5	− 4.3	− 2.5
Molecular weight	882.68		–	–	–	–
Flashpoint (°C)	–	126	68	112	118	124
Iodine value (gI_2_/100 g)	103.04		–	–	–	–
Pour point (°C)	− 3	− 2.1	− 17.5	− 8.6	− 7.2	− 5.5
Moisture content (%)	0.55		–	–	–	–
pH	6.8	7.54	–	–	–	–
Cetane number	–	52.1	48.7	49.6	51.2	51.6

### Engine performance analysis

#### Brake specific fuel consumption (BSFC)

**Fig. 3:**
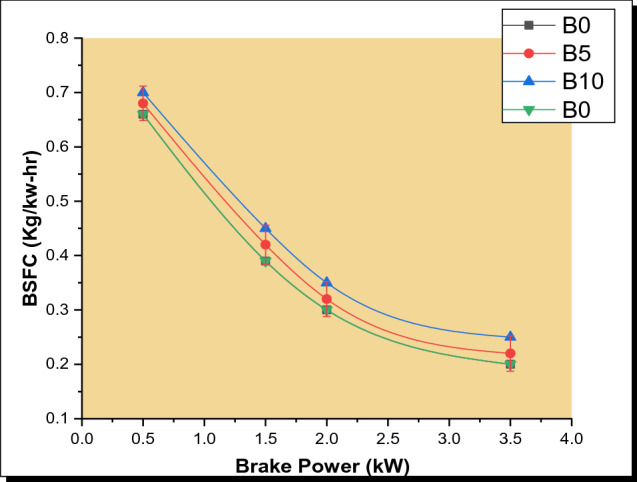
Plot of BSFC with BP for various PSB blends

The brake-specific fuel consumption for the different parsley biodiesel blends is shown in Fig. 3. The BSFC of diesel, as well as varied biodiesel blends, showed a declining pattern with a rise in brake power (BP). The decrease in BSFC could be a result of the increased engine’s workload which gives a lower fuel consumption rate that necessitates higher power [34]. The obtained result revealed that B0 had the least BSFC and BSFC increased as the proportion of biodiesel in the blend increased. This is similar to the observations of Elkelawy et al. [9] and Ogunkunle and Ahmed [8]. In comparison to diesel, a slight increase of about 1.55%, 2.77%, and 3.2%, were observed for the respective biodiesel blends of B5, B10, and B20. Several researchers have reported that the low calorific value, as well as high density of biodiesel, is attributable to its increased BSFC as the biodiesel blend ratio is increased [35]. Moreover, parsley oil has a greater density than diesel fuel, resulting in increased consumption. In addition, as previously reported by Atabani et al. [36], the increased kinematic viscosity of biodiesel blends could contribute to poor fuel atomization which leads to the poor blending of fuel with air and eventually a rise in BSFC. More so, the low engine load conditions necessitate a higher BSFC, since the cylinder temperature declines more than the engine load, and incomplete fuel combustion results [37].

#### Brake thermal efficiency (BTE)

**Fig. 4:**
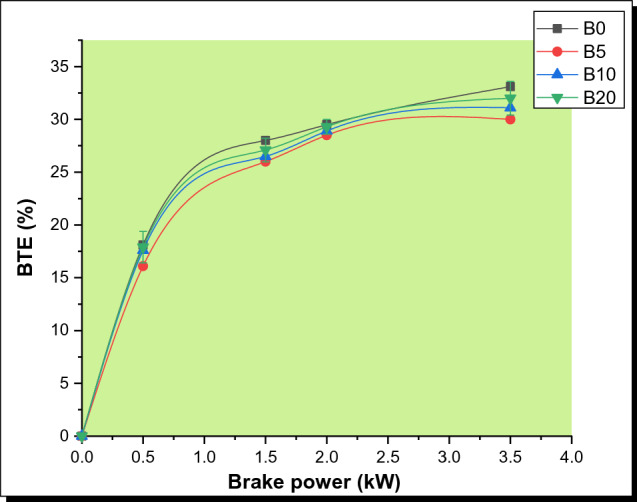
Plot of BTE with BP for various PSB blends

Figure 4 shows the brake thermal efficiency for the four various test fuels evaluated. For all of the fuel samples tested, the BTE increases as the load increases. A reduction in heat loss, as well as a rise in power as the percentage load increases, can be ascribed to the increase in BTE. For B0, B5, B10, and B20 test samples, the optimum brake thermal efficiency obtained at full load was 33.1%, 30.0%, 31.1%, and 32.0%, respectively. The increase in thermal efficiency observed with the biodiesel samples can be linked to their lower calorific value, which leads to a higher fuel supply for a given load, resulting in shorter combustion times and negating the potential benefit of fuel-borne oxygen in boosting the combustion process [35]. The findings are consistent with the findings of other researchers Banapurmath et al. [38, 39]. In addition, biodiesel samples have a higher viscosity and lower power, which cause a drop in brake thermal efficiency. Biodiesel's low volatility and vaporization properties produce a non-uniform mixture in the combustion chamber during atomization which may favor incomplete combustion [40].

#### Brake specific energy consumption (BSEC)

**Fig. 5:**
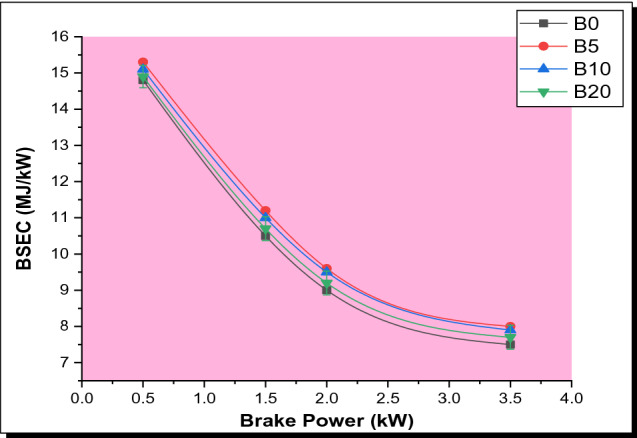
Plot of BSEC with BP for various PSB blends

Brake specific energy consumption as a function of brake power for various test samples analyzed is depicted in Fig. 5. BSEC is a dependable element for evaluating various fuels with respect to their different heating values. The utilization of BSEC is very vital to perfectly understand the effectiveness of converting its energy content into useable power [41]. It was observed that the BSEC decreases as the load increases. The explanation for the dramatic drop is that under higher loads, the amount of fuel needed to run the engine per unit energy output drops [42]. When comparing all the biodiesel blends to pure diesel, it was discovered that BSEC is higher for all blends, although the BSEC decreases as the proportion of the biodiesel increases. It is also worth noting that engine power is affected by both torque and speed. Torque can be raised to increase power. Since the stored energy in the fuel is easily liberated via combustion at reduced speeds, energy is expended at lower torques [43]. In addition, since biodiesel blends possess greater atoms of carbon in comparison with parsley biodiesel, the combined fuel (blends) combust much faster and consume greater energy at lower torque, diminishing energy consumption efficiency as power increases.

### Engine exhaust emission analysis

#### Nitrogen oxides (NO_*x*_) emissions

**Fig. 6:**
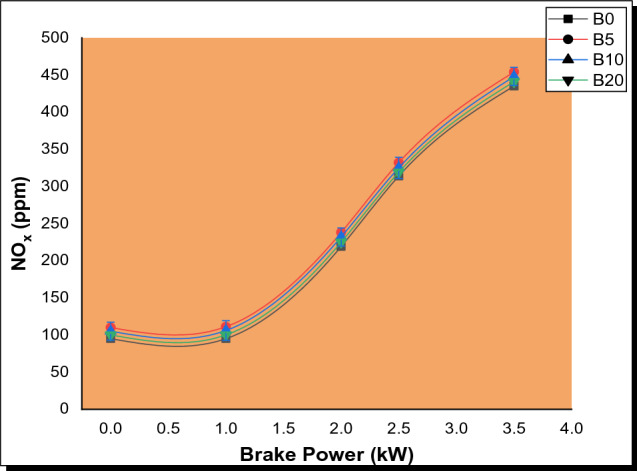
Variation of NO_*x*_ with brake power for PSB blends

The NO_*x*_ emission of Parsley biodiesel blends was tested at various load levels as depicted in Fig. 6. At all loads, NO_*x*_ emissions from parsley biodiesel blends were found to be greater than those from pure diesel. The NO_*x*_ emissions on average increased by 20.48%, 20.00%, and 19.50% for B5, B10, and B20, respectively. This is because the NO_*x*_ emissions from biodiesel blends are greatly influenced by fuel properties, engine load, and oxygen concentration [44]. The slight increase in NO_*x*_ emissions observed with parsley biodiesel blends can be attributed to the high oxygen content of biodiesel fuel. In addition, the utilized biodiesel fuel has more unsaturated fatty acids, which have a higher adiabatic flame temperature, resulting in higher NO emissions [45]. The obtained result is similar to the findings of a few researchers who claimed that biodiesel blends emit more NO_*x*_ than traditional diesel fuels [46, 47].

#### Carbon monoxide (CO) emissions

**Fig. 7:**
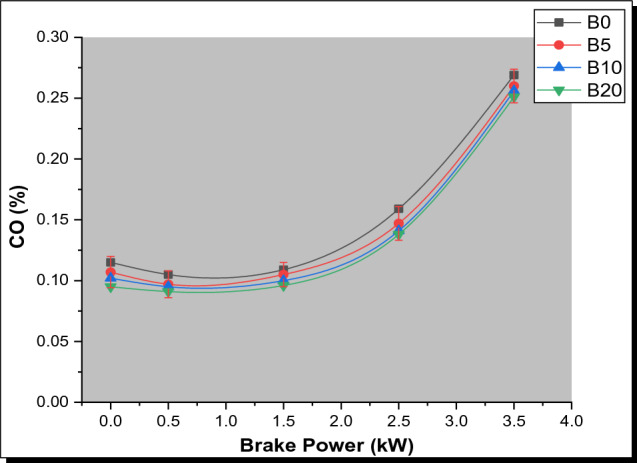
Variation of CO with brake power for PSB blends

Figure 7 shows the CO emissions for PSB and diesel blends as a function of BP variation. The CO emissions were low at small and medium loads (for the test fuel samples) and then a gradual increase in CO emission was observed (at maximum load) as the brake power rises. This occurred because of differences in air–fuel input throughout the engine e’s various operating conditions [48]. Moreover, increased engine loads result in increased CO emissions as a result of more fuel being burned and the production of much more fuel-rich zones with low O_2_ levels [49]. It was seen that the CO emissions of the produced biodiesel blend (B20) were quite similar to that of diesel. This is attributable to the abundance of oxygen in biodiesel which favors complete combustion. Even when all the prepared parsley biodiesel blends were compared to diesel, all biodiesel blends exhibited a decline in CO emissions. On average, the CO emissions were reduced by 28.4%, 31.6%, and 33.9% for B5, B10, and B20, respectively. In addition, CO emissions reduced as the amount of parsley biodiesel in the blends increased, and this effect was most noticeable in B20 blends. The excess air ratio during combustion as parsley biodiesel level rises is associated with a decrease in CO emissions [50]. In comparison to diesel, the cylinder possesses an abundance of oxygen for combustion which in turn aids the oxidation of much more molecules of carbon. B20 Parsley biodiesel blend had the least carbon monoxide emission when compared to all the test biodiesel blends. Furthermore, the experimental result obtained has similar results outputs derived from previous experimental outcomes [51, 52].

#### Hydrocarbon (HC) emissions

**Fig. 8:**
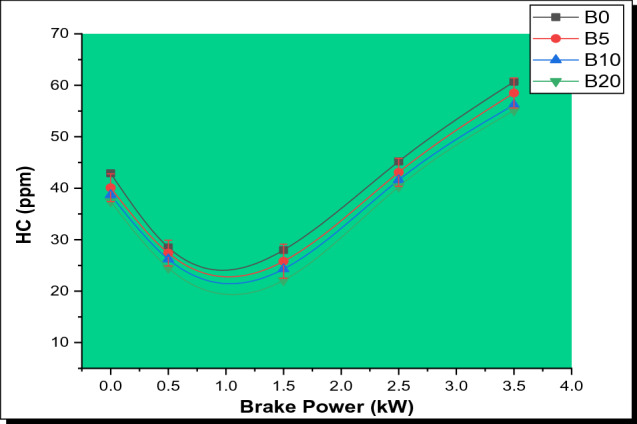
Variation of HC with brake power for PSB blends

Figure 8 shows the fluctuation in HC emission as a function of BP. The graph revealed that the amount of HC emissions decreased somewhat as the biodiesel percentage increased. All blends have reduced hydrocarbon emissions at partial load, although they rise at full load. This is owing to the fact that when more fuel is pumped into the cylinder under a greater load, there is less oxygen available for the reaction. However, B5, B10, and B20 decreased by 6.91%, 9.32%, and 11.38%, respectively, in comparison to pure diesel. HC emission of B20 possesses the least emissions in comparison to other test samples. HC emissions are reduced when the blend ratio rises from B5 to B20, since the ester-based fuels have a greater cetane number than diesel. In addition, the lower HC emission is the result of a shorter latency time. Furthermore, when the biodiesel blend ratio increases, the intrinsic oxygen possessed by the esters plus rising oxygen concentration (a rise in the amount of parsley biodiesel in the blend) results in improved combustion, leading to a reduction in HC emission [39]. The HC emission levels recorded in this experiment were found to be similar to those found in earlier studies [49, 53].

#### Carbon dioxide (CO_2_) emissions

**Fig. 9:**
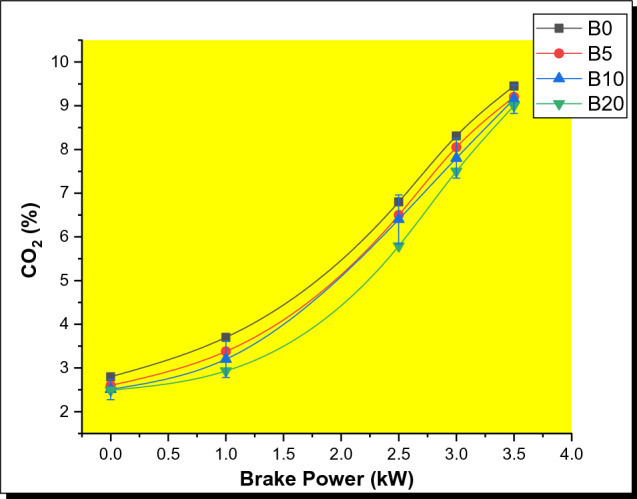
Variation of CO_2_ with brake power for PSB blends

The variations of CO_2_ emissions with engine load for diesel and biodiesel–diesel blends, are shown in Fig. 9. An increasing trend in CO_2_ emissions was seen for all test samples as the brake power increases. However, the study found that increasing the volume proportion of biodiesel in diesel results in a decline in CO_2_ emissions. The CO_2_ emissions were reduced by 29.06%, 29.67%, and 29.73% for B5, B10, and B20, respectively, compared to diesel. This is associated with the biodiesel's oxygen abundance which allows for a longer and more stable diffusion combustion phase [54]. Nonetheless, the amount of CO_2_ generated by the blends is more than the amount of CO emitted. It is vital to understand that since biodiesel possesses an abundance of oxygen molecules, excess oxygen available in the blends favors complete combustion. Thereby contributing to the reaction between oxygen and carbon to yield CO_2_. Reduction in the amount of oxygen has a substantial impact on the production of carbon monoxide in engines. However, the B0 sample possessed the highest CO_2_ emissions, while the B20 blend had the lowest emission.

## Conclusions

The following observations can be made from the study based on the findings obtained and the analysis performed:

- An alkali methanolysis was found to be successful in the synthesis of biodiesel from Parsley seed oil with the fuel characteristics meeting ASTM D6751 criteria.
- B20 has fuel properties that are somewhat similar to that of diesel.
- According to the results of the investigation, B20 blended fuel does have the best fuel ratio when compared to other blends in terms of improving engine performance and emission characteristics.
- BSFC increased as the proportion of biodiesel in the blend increased. In comparison to diesel, a slight increase of about 1.55%, 2.77%, and 3.2%, were observed for biodiesel blends of B5, B10, and B20.
- For all of the fuel samples tested, the BTE increased as the brake power increases For B5, B10, and B20 test samples, the optimum brake thermal efficiency obtained at full load was 30.0%, 31.1%, and 32.0%, respectively.
- BSEC was higher for all blends (B5 B10 and B20) compare to pure diesel (B0). However, BSEC reduces as the biodiesel proportion increases. In addition, BSEC decreases as the load increases for all test samples.
- For the biodiesel blends, it was observed that the emissions of HC decreased by 6.91%, 9.32%, and 11.38% for B5, B10, and B20. In addition, CO emissions were reduced by 28.4%, 31.6%, and 33.9% for B5, B10, and B20, respectively.
- CO_2_ emissions showed an increasing trend for all samples but compared to diesel, CO_2_ emissions were reduced by 29.73% for the B20 blend.
- There was an increase in emission of NO_*x*_ emissions compared to diesel fuel. The NO_*x*_ emissions on average increased by 20.48% 20.00% and 19.50% for B5, B10, and B20, respectively.
- Biodiesel from Parsley is suitable as an alternate fuel for diesel engines for a green environment.
